# Corrosion Resistant Multi-Principal Ni_60_Cr_15_Co_15_Ti_5_Al_2.5_Nb_2.5_ Alloy: Loss of Passivation (LOP) and Recovery of Passivation (ROP) upon Heat Treatment

**DOI:** 10.1021/acsomega.6c08640

**Published:** 2026-09-11

**Authors:** Virgilio Pereira Ricci, Emerson Cortez Gallego Campos, Victor Hugo Mafra Monfredo Ferreira, Rafael Magalhães Triani, Guilherme Zepon, Francisco Gil Coury, Eric Marchezini Mazzer, Guilherme Yuuki Koga

**Affiliations:** † Department of Materials Engineering (DEMa), 67828 Federal University of São Carlos (UFSCar), Rodovia Washington Luís, km 235 SP-310, 13565-905 São Carlos, SP, Brazil; ‡ Institute of Innovation in Advanced Materials, National Industrial Training Service (SENAI), Rua Vitória Médici Ramos, CEP 09861-790, São Bernardo do Campo, São Paulo, Brazil; § Center for Characterization and Development of Materials (CCDM), Federal University of São Carlos (UFSCar), Rod. Washington Luís, CEP 13565-905 São Carlos, SP, Brazil

## Abstract

This study explores the corrosion behavior of a nonequiatomic Ni_60_Cr_15_Co_15_Ti_5_Al_2.5_Nb_2.5_ high-entropy alloy (HEA) subjected to thermal aging. Electrochemical testing in 3.5 wt% NaCl solution revealed that short-term aging (≤1 h) induces a loss of passivation (LOP), marked by a corrosion potential drop to −260 mV_SCE_ and an increase in corrosion current density *i*_corr_ to 2.7 μA/cm^2^. In contrast, prolonged aging (≥10 h) promotes a recovery of passivation (ROP), driven by microstructural elemental redistribution. This recovery is evidenced by a positive shift in corrosion potential to −125 mV_SCE_, a significant reduction in *i*_corr_ to 7.1 × 10^–2^ μA/cm^2^, and an increase in pitting potential (*E*_pit_) from +413 mV_SCE_ to +495 mV_SCE_. Electrochemical impedance spectroscopy (EIS) further confirmed enhanced passivity through a reduction in effective capacitance (from 3.2 to 2.6 μF/cm^2^) and an increase in polarization resistance (from 960 to 1084 kΩ·cm^2^). However, excessive aging (>50 h) may trigger a secondary LOP. An optimal aging window (30–50 h) yields a stable and protective passive film.

## Graphical abstract

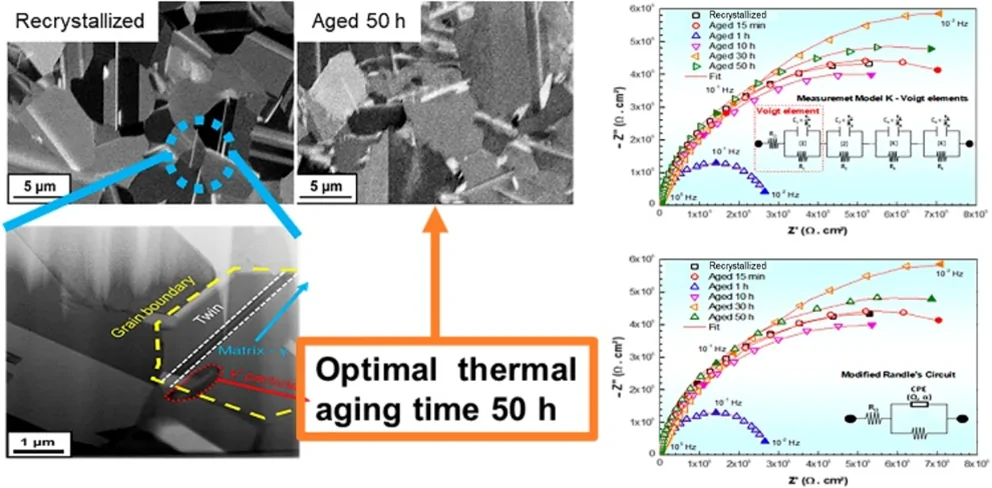

## 1 Introduction

High-entropy alloys (HEAs), also referred to as multi-principal element alloys (MPEAs), constitute a transformative class of materials characterized by the inclusion of five or more principal elements in near-equiatomic or equiatomic ratios. This unconventional alloy design strategy disrupts traditional metallurgy by promoting the formation of stable single-phase or multiphase solid solutions, rather than intermetallic compounds. As a result, some HEAs can exhibit interesting combination of properties, including high mechanical strength, thermal stability, and corrosion resistance. These attributes make HEAs highly attractive in the search for structural and functional applications in extreme environments such as aerospace, nuclear energy systems, and marine engineering.^1,2^

Unlike conventional alloys, such as stainless steels or aluminum alloys, where corrosion resistance relies heavily on minor additions of elements like Cr or Al to form protective layers, HEAs can exhibit resistance to degradation due to their complex concentrated composition.^2–4^ This inherent resistance arises from synergistic interactions among alloying elements, which can promote the formation of dense, adherent passive films that act as barriers against corrosive media.^5–7^

Among the critical attributes of high-entropy alloys (HEAs), corrosion resistance has emerged as a key focus, especially for applications in chemically aggressive or extreme environments. Central to this resistance is the formation and stability of a passive film, which normally corresponds to a nanoscale oxide layer that spontaneously develops on the alloy surface, acting as a protective barrier against further degradation. The effectiveness of this passive layer is strongly influenced by the alloy’s chemical composition. In particular, elements such as chromium (Cr), niobium (Nb), and aluminum (Al) contribute significantly to passivation by forming stable, adherent oxides (e.g., Cr_2_O_3_, Nb_2_O_5_) or by strengthening the overall oxide thin film.^8,9^

Thermal exposure further influences corrosion performance by driving microstructural evolution and/or distribution or partition of the elements. On the positive side, this can enhance resistance through mechanisms such as enhanced elemental homogenization (e.g., Al, Cu, Mo, Cr) and suppression of detrimental phase segregation.^8,9^ Of particular importance is the role of Nb, which has been shown to improve passivity either by reinforcing the oxide framework of other elements^10^ or by forming a robust, Nb_2_O_5_-containing passive film that increases the alloy’s durability in corrosive media.

Passivation is thus a fundamental corrosion protection mechanism,^11,12^ which can be compromised under harsh thermal or chemical conditions, leading to Loss of Passivation (LOP). Conversely, Recovery of Passivation (ROP) may occur through thermally induced microstructural reconfiguration that restores or enhances the protective oxide layer. A comprehensive understanding of these dynamic passivation behaviors is essential for predicting long-term alloy performance and ensuring structural integrity in demanding environments.^13^

Despite extensive research on HEAs, the LOP and ROP remain poorly understood, particularly in the context of nonequiatomic compositions. Most studies have focused on equiatomic systems, resulting in limited understanding of how compositional deviations from equiatomic ratios, such as in the Ni-rich alloy (Ni_60_Cr_15_Co_15_Ti_5_Al_2.5_Nb_2.5_), affect passivation behavior.^14–16^ Furthermore, the transient degradation of passive films during early stages of heat treatment and their subsequent recovery with prolonged aging has not been systematically investigated. Addressing these knowledge gaps is critical for tailoring HEAs to real-world applications where fluctuating thermal and chemical conditions are prevalent.^15^

In this study, the passivation and depassivation behavior of a nonequiatomic Ni_60_Cr_15_Co_15_Ti_5_Al_2.5_Nb_2.5_ HEA were evaluated, with a particular focus on the role of thermal aging in modulating passive film stability. Using electrochemical techniques including cyclic polarization and electrochemical impedance spectroscopy (EIS), we demonstrated that short-term aging leads to a transient loss of passivation, while extended thermal exposure facilitates the recovery of a passive film. By elucidating the loss and recovery of passivation in nonequiatomic HEAs, this work advances our understanding of the corrosion behavior in Ni-based complex concentrated alloys.

A version of this study was previously published.^17^ During a subsequent review of the work, the authors identified that the structural characterization, particularly the phase identification and associated discussion, required more in-depth analysis to ensure the accuracy and clarity of the interpretation. The manuscript was therefore revised. The present article supersedes the previous publication.

## 2 Results and Discussion

### 2.1 Alloy Design

Figure 1 shows the isopleth sections calculated at 750 °C. Figure 1a shows the Al–Nb isopleth, where Nb is progressively added at the expense of Al, while Ti is kept constant at 5 at%. Figure 1b shows the Al–Ti isopleth, where Ti is progressively added at the expense of Al, while Nb is kept constant at 2.5 at%. Using the isopleth section of Al–Nb and Al–Ti, it is possible to evaluate the formation of the strengthening phases L1_2_ (γ′) and D0_22_ (γ″) at the aging temperature of 750 °C for the multicomponent alloy Ni_60_Cr_15_Co_15_Ti_5_Al_2.5_Nb_2.5_ (mol%). Both diagrams validate the compositional strategy of the alloy, where the combined presence of Al, Ti, and Nb promotes the desired phase equilibrium at the applied aging temperatures. Qiao et al. explained that when the Al/Ti ratio is around 1:1, γ′ forms first, creating Nb-rich zones that favor γ″ nucleation, resulting in γ′/γ″ coprecipitates.^18^

**Figure 1:**
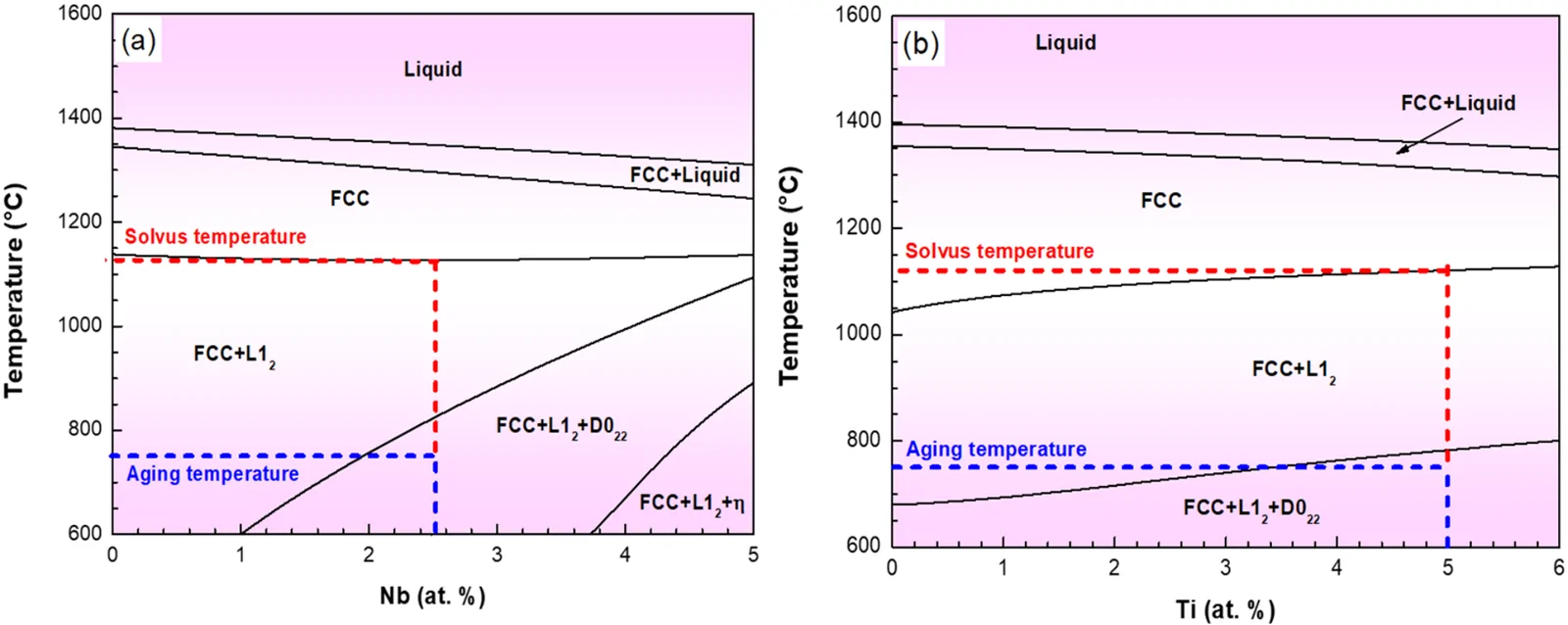
Calculated isopleth sections of the NiCrCoAlTiNb system at 750 °C: (a) Al–Nb isopleth section, showing the progressive substitution of Al by Nb (Nb fixed at 2.5 at%); (b) Al–Ti isopleth section, showing the progressive substitution of Al by Ti (Ti fixed at 5 at%). The position of the target alloy Ni_60_Cr_15_Co_15_Ti_5_Al_2.5_Nb_2.5_ is highlighted in both diagrams, confirming its stability within the FCC + γ′ + γ″ region. The used database was PanNi2022.

At a 2:1 ratio, more γ′ forms due to increased Al, but Nb dissolves into γ′, reducing γ″ formation. At 3:1, excess Al lowers Nb in γ′, enabling new γ″ precipitation at aging temperatures. Thus, the Al/Ti ratio and Nb content critically control γ′ and γ″ phase evolution during aging.^18^ In the Al–Nb diagram, Figure 1a, the composition corresponding to 2.5 mol% Nb and 2.5 mol% Al is located within the FCC + L1_2_ + D0_22_ phase field at 750 °C, confirming the simultaneous stability of both ordered phases. Niobium acts as the primary stabilizer of the D0_22_ phase.

Similarly, the Al–Ti isopleth section (Figure 1b) shows that at 5 mol% Ti and 2.5 mol% Al, the alloy composition also falls within the FCC + L1_2_ + D0_22_ region at 750 °C, indicating that titanium promotes the stability of the L1_2_ phase. The specific ratios of aluminum, niobium, and titanium critically influence the phase boundaries and stability within the FCC matrix, enabling the coexistence of the L1_2_ and D0_22_ phases at the selected aging temperature. Although the theoretical High-Throughput Calculations (HTC) based calculations initially estimated the solvus temperature at 1089 °C, projection of the phase diagrams using the real alloy composition indicated a revised solvus temperature of 1125 °C, which still preserves the desired FCC + L1_2_ + D0_22_ fields. This adjustment further confirms the robustness of the design strategy.

For this reason, 750 °C was selected as the aging treatment temperature, as it not only matches the equilibrium condition of FCC + L1_2_ + D0_22_ for the actual alloy composition but is also widely reported in the literature as the typical aging temperature for nickel-based superalloys operating in high-temperature environments.

### 2.2 Microstructure Analysis

Backscattered electron (BSE) imaging via scanning electron microscopy (SEM) revealed a fine, equiaxed grain structure (∼5 μm) in the recrystallized state (Figure 2a), consistent with a single-phase microstructure as reported in prior studies.^12,19,20^ Even prolonged aging heat treatments (15 min to 50 h) induced only marginal grain growth, maintaining an average grain size of ∼5 μm, Figure 2b–f, suggesting the presence of grain-boundary pinning mechanisms (e.g., solute drag or secondary-phase stabilization). Heat treatment from 15 min to 50 h caused slightly grain growth to ∼8 μm.

**Figure 2:**
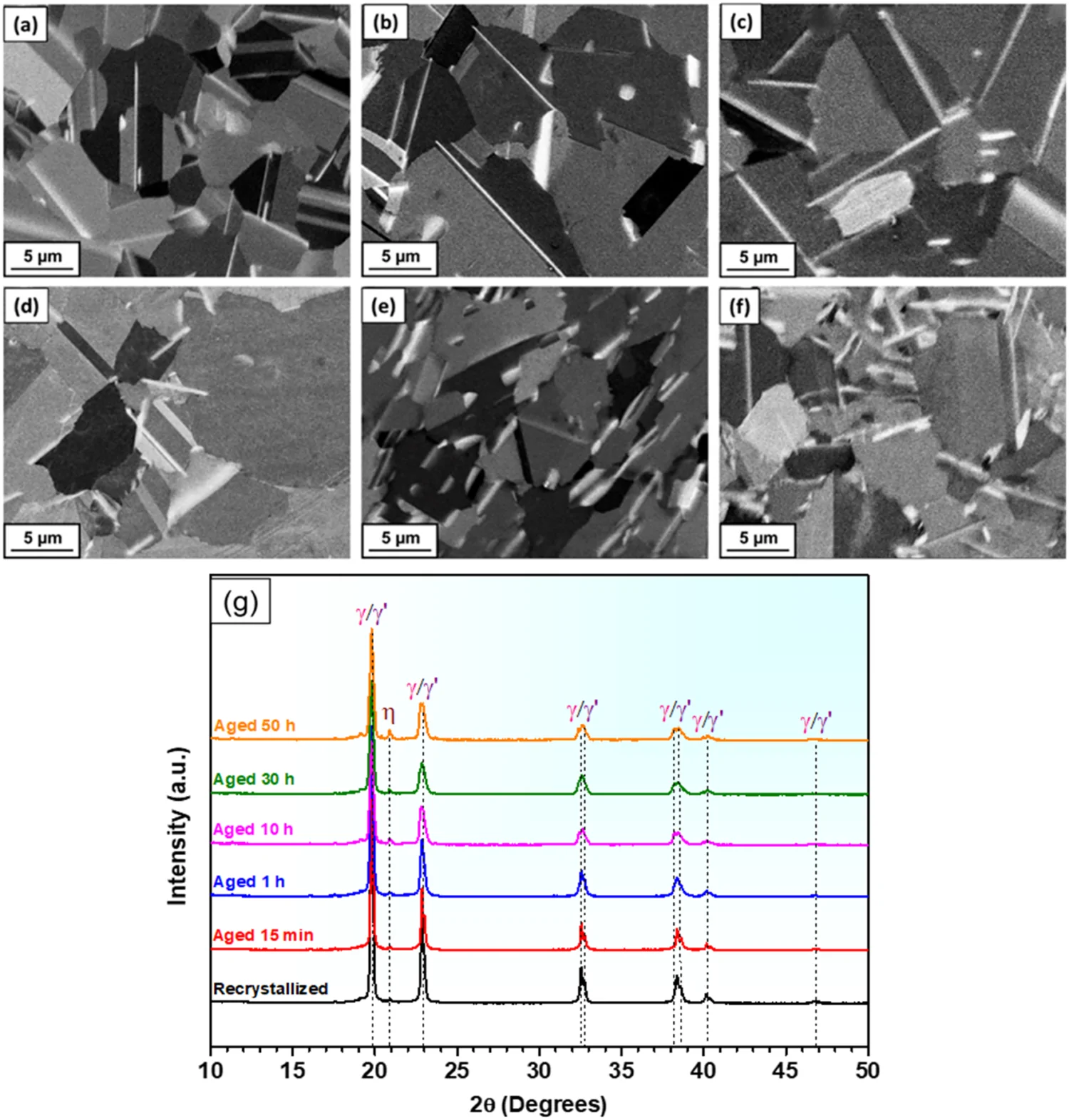
(a–f) SEM micrographs acquired in backscattered electron (BSE) mode showing the microstructural evolution of the HEA under different thermal aging conditions: (a) recrystallized, (b) aged for 15 min, (c) aged for 1 h, (d) aged for 10 h, (e) aged for 30 h, and (f) aged for 50 h. (g) Corresponding X-ray diffraction (XRD) patterns highlighting phase evolution across the aging duration.

X-ray diffraction (XRD) analysis (Figure 2g) confirmed the dominance of the γ-phase (FCC solid solution) in the as-processed state. With increasing aging times, additional diffraction peaks emerge, which are consistent with the formation and growth of the η (Ni_3_Ti-type) intermetallic phase. The identification of the γ′ phase from XRD alone remains ambiguous, since the fundamental reflections of γ and γ′ largely overlap and the expected superlattice reflections of L1_2_ exhibit very low intensity under the present experimental conditions but it also present on the material as will be shown by transmission electron microscopy (TEM).

Coarse precipitates preferentially nucleate at grain boundaries with increasing aging time (Figure 2b), exhibiting morphologies consistent with the η intermetallic phase observed in the SEM micrographs. With prolonged aging, the increased fraction and contrast of these precipitates indicate progressive precipitation and coarsening with aging time, which is consistent with the increase in the η peak in XRD patterns shown previously. The γ-matrix remains the dominant phase, while the evolution of secondary intermetallic phases, particularly η, is expected to significantly affect the mechanical response. In Ni-based alloys, the η phase is generally regarded as deleterious,^21–23^ especially when forming coarse or continuous grain-boundary precipitates, as it tends to reduce ductility and fracture resistance rather than provide effective precipitation strengthening. The limited grain growth observed after extended aging suggests an effective grain-boundary pinning effect by these secondary phases.

The representative energy-dispersive X-ray spectroscopy (EDS) values in at% obtained in Figure 2a,e, recrystallized and aged 50 h conditions, respectively, are shown in Table 1. The small variation in measurements indicates a very homogeneous chemical composition across the samples. The results also show that the alloy, under different heat treatment conditions, exhibits a chemical composition close to the desired one.

**Table 1:** EDS Results Performed in Figure 2a,e, Recrystallized and Aged 50 h Conditions

	conditions	
elements (at%)	recrystallized	aged 50 h
Nickel	59.47	59.70
Chromium	14.83	14.49
Cobalt	15.62	15.76
Titanium	5.15	5.18
Aluminum	2.87	2.67
Niobium	2.06	2.20

Qualitatively, the γ (FCC) phase remains the dominant constituent under all aging conditions, while the secondary intermetallic phase that develops is consistently associated with the η (Ni_3_Ti-type). Accordingly, changes in XRD peak intensity and peak broadening are more consistently interpreted as variations in the volume fraction, size, and coherency of η precipitates, instead of bulk texture reorientation.

Figure 3a,b provide a high-resolution investigation of the microstructural and compositional features in the recrystallized condition of the Ni_60_Cr_15_Co_15_Ti_5_Al_2.5_Nb_2.5_ alloy, prior to thermal aging. This analysis, conducted via Scanning Transmission Electron Microscopy in Bright Field (STEM-BF), Selected Area Electron Diffraction (SAED), and STEM-EDS mapping, is essential for understanding early stage precipitation and elemental partitioning, even in the absence of prolonged aging. From a partitioning standpoint, the precipitation of the Ni–Ti-rich η phase is expected to redistribute primarily Ti, Cr, and Co from the matrix, as shown in Figure 3.

**Figure 3:**
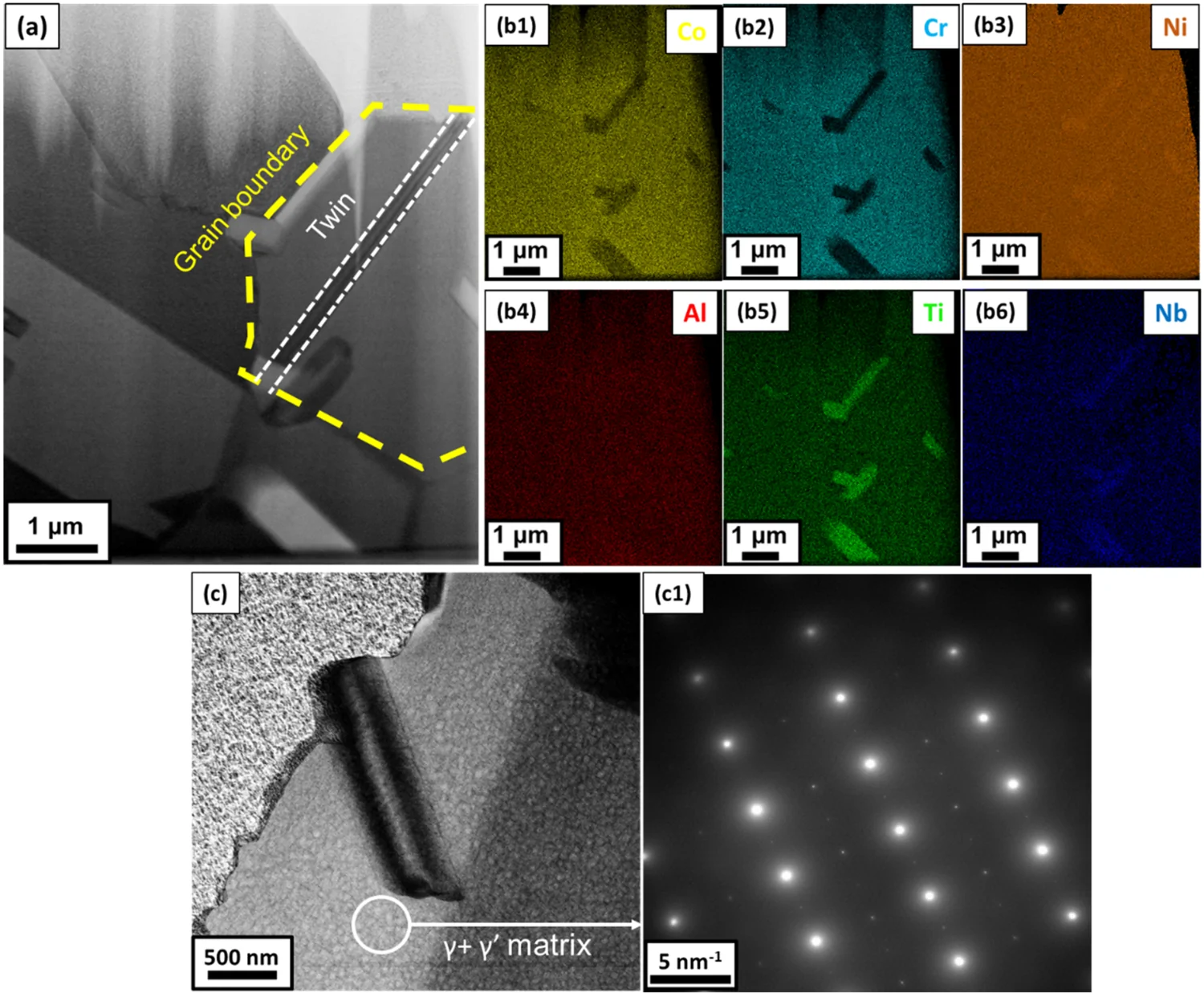
(a) STEM-BF image showing η precipitates within the FCC-γ matrix in the recrystallized condition. (b1–b6) STEM-EDS elemental maps showing the spatial distribution of Co (b1), Cr (b2), Ni (b3), Al (b4), Ti (b5), and Nb (b6), demonstrating elemental partitioning between the η precipitates and the surrounding matrix. (c1) STEM-BF image revealing a γ + γ′ matrix as shown in [112] selected area diffraction pattern where the γ′ superlattice reflections.

The STEM-BF (Figure 3a) micrograph reveals a heterogeneous nanoscale microstructure, with contrast variations indicative of incipient precipitation within the FCC γ matrix. The nanometric features embedded in γ exhibit morphologies consistent with the η intermetallic phase. Importantly, the STEM-EDS maps in Figure 3b show that these coarse intergranular features are enriched in Ni, Ti and Nb, which is consistent with the expected chemistry of the η (Ni_3_Ti-type) phase. Cr also appears more uniformly distributed or slightly depleted in the precipitates, aligning with its preference to remain in the γ/γ′ matrix and contribute to corrosion resistance via Cr_2_O_3_ formation. Co is also evenly distributed, with slight preference for the matrix.

The STEM-BF (Figure 3c1) and the selected area electron diffraction pattern in Figure 3c2 collected from the γ matrix along the [112] zone axis display a typical cubic symmetry, consistent with an FCC structure. Superlattice reflections can be seen at the 1/2 {220} reflections, consistent with {110} superlattice reflections present on a γ + γ′ mixture. The inhomogeneous contrast on the matrix reinforces the nanometric size of the precipitates. Weak reflections in the 1/2 {111} position can also be seen and those cannot be attributed to γ′, they are likely originated from a thin and crystalline Cr_2_O_3_ oxide on the surface of the sample that could generate these additional reflections given the orientation relationship published in the literature between these phases.^24^

The early presence of γ′ precipitates indicate that the system is primed for precipitation hardening. Furthermore, the spatial distribution of corrosion-relevant elements (Cr, Nb) throughout the matrix suggests that passive film formation may be localized, depending on the surface exposure of γ vs η regions.

The precipitation of the γ′ phase (Ni_3_(Al,Ti)) in Ni-based high-entropy alloys is governed by a delicate interplay between thermodynamic driving forces and kinetic parameters such as solute diffusivity and interfacial energy. In multicomponent systems like Ni_60_Cr_15_Co_15_Ti_5_Al_2.5_Nb_2.5_, the supersaturation of Al and Ti within the FCC matrix promotes the early nucleation of γ′, a phenomenon accentuated by the low lattice mismatch (∼0.2–0.5%) with the γ-phase, which reduces the interfacial energy barrier for coherent precipitation.^22,25^

The partitioning of Ti and Nb into η and the exclusion of elements like Co and Cr from these domains align with classical partitioning behavior in Ni-based systems, where Nb typically remains in solid solution or forms distinct secondary phases at longer aging times.^18,26^ Importantly, the η precipitates contribute significantly to microstructural stability by exerting Zener drag on migrating grain boundaries, effectively restricting grain growth during thermal exposure.

High-resolution STEM-EDS mapping and SAED analyses confirmed the elemental partitioning and provided direct evidence of η precipitates (Ni_3_(Ti,Nb,Al)) enriched in Nb and Ti, in agreement with the XRD patterns. The persistence of Cr and Nb in the γ matrix is particularly relevant, as these elements contribute to the passive stability of the film through the formation of protective oxides, such as Cr_2_O_3_ and Nb_2_O_5_. Furthermore, the limited grain growth observed even after 50 h of aging indicates the Zener pinning action of Nb/Ti-rich precipitates, which preserve fine grain size and directly influence the mechanical and corrosion performance of the alloy.

### 2.3 Electrochemical Corrosion

The corrosion behavior of the Ni_60_Cr_15_Co_15_Ti_5_Al_2.5_Nb_2.5_ HEA under different thermal treatments was investigated through cyclic polarization testing in 3.5 wt% NaCl solution. The cyclic polarization curves (Figure 4) and corresponding electrochemical parameters (Table 2) reveal insights into the effects of heat treatment on corrosion behavior.

**Table 2:** Electrochemical Parameters of Ni_60_Cr_15_Co_15_Ti_5_Al_2.5_Nb_2.5_ Extracted from the Cyclic Polarization Curves Shown in Figure 4 under Various Thermal Aging and Recrystallized Conditions

samples	*E*_corr_ (mV_SCE_)	*i*_corr_ (μA/cm^2^)	*i*_pass_ (mA/cm^2^)	*E*_pit_ (mV_SCE_)	*E*_prot_ (mV_SCE_)
Recrystallized	–305 ± 15	2.6 ± 0.05	1.6 ± 0.02	+413 ± 25	–56 ± 6
Aged 15 min	–173 ± 8	0.8 ± 0.02	0.2 ± 0.07	+351 ± 21	–106 ± 8
Aged 1 h	–260 ± 13	2.7 ± 0.03	0.5 ± 0.03	+5 ± 19	–227 ± 14
Aged 10 h	–285 ± 11	2.1 ± 0.01	0.3 ± 0.06	+300 ± 15	–209 ± 11
Aged 30 h	–176 ± 9	0.9 ± 0.01	4.0 ± 0.04	+480 ± 17	289 ± 10
Aged 50 h	–125 ± 10	0.07 ± 0.001	0.3 ± 0.03	+495 ± 32	–41 ± 9

**Figure 4:**
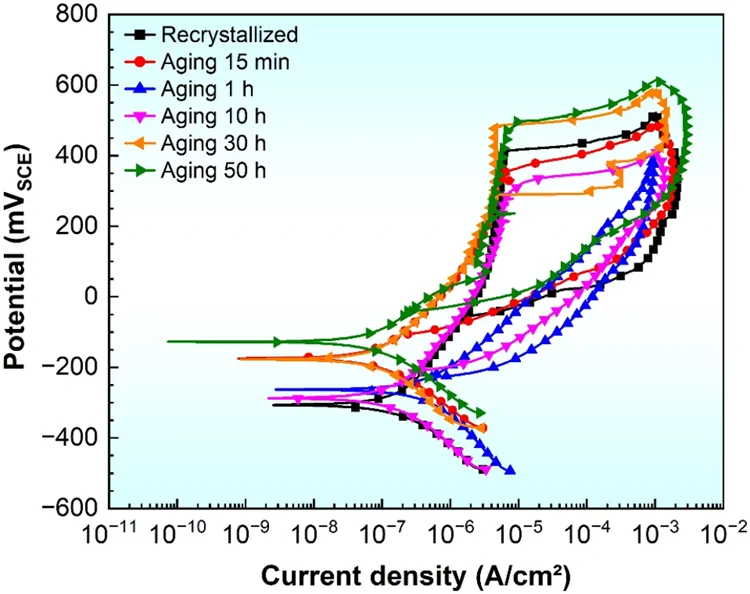
Cyclic polarization curves of the Ni_60_Cr_15_Co_15_Ti_5_Al_2.5_Nb_2.5_ HEAs under different thermal aging conditions, tested in 3.5 wt% NaCl solution.

To ensure clarity in the interpretation of the electrochemical behavior, key parameters used throughout this section are herein defined. The corrosion potential (*E*_corr_) corresponds to the potential at which the anodic and cathodic currents are balanced and the net current is zero. The corrosion current density (*i*_corr_) is extracted by Tafel extrapolation of the cathodic branch and provides an index related to the corrosion rate. The passivation current density (*i*_pass_) denotes the relatively stable current density within the passive region, indicating the effectiveness of the passive film in limiting anodic dissolution. The pitting potential (*E*_pit_) represents the potential at which a sharp increase in current density occurs, marking the onset of localized breakdown of the passive film. Conversely, the protection potential (*E*_prot_) is determined during the reverse scan, where a distinct drop in current signals the reformation of a passive film. These electrochemical parameters collectively enable a comprehensive assessment of passivation stability, susceptibility to localized corrosion, and the influence of thermal treatment on passive film integrity.

The as-recrystallized sample exhibited the most negative *E*_corr_ (−305 mV_SCE_). With increasing aging time, *E*_corr_ shifted positively, reaching −125 mV_SCE_ after 50 h, indication of enhanced passivation and reduced corrosion tendency.^11^ This positive shift highlights a progressive ROP due to the formation of a more protective oxide film. Conversely, a marked drop to −260 mV_SCE_ after 1 h of aging reflects a transient LOP, likely due to microstructural instability or partial oxide disruption during early stages of thermal exposure.

Corroborating the *E*_corr_ trend, the corrosion current density (*i*_corr_) decreased significantly with aging. The *i*_corr_ of the recrystallized sample was 2.6 × 10^–6^ A/cm^2^, whereas after 50 h of aging it decreased to 7.1 × 10^–8^ A/cm^2^, indicating more than an order-of-magnitude improvement in corrosion resistance.^12,16^ The *i*_corr_ after 1 h of aging (2.7 × 10^–6^ A/cm^2^) resembles that of the as-recrystallized sample, but passivation was no longer observed, suggesting a brief LOP event during initial aging. These findings demonstrate that thermal exposure initially causes LOP but subsequently enables the development of a stable and adherent passive film (ROP) after prolonged aging, markedly enhancing corrosion resistance.

The passivation current density (*i*_pass_) also decreased substantially with prolonged aging. Initially high in the recrystallized state (1.6 × 10^–3^ A/cm^2^), *i*_pass_ declined to 2.5 × 10^–4^ A/cm^2^ after 50 h, confirming ROP and the maturation of a denser and more protective passive layer. Interestingly, a temporary increase in *i*_pass_ after 30 h (4.0 × 10^–3^ A/cm^2^) suggests possible passive film breakdown or pore formation, contributing to transient LOP prior to full recovery with extended aging.

The pitting potential (*E*_pit_) followed a similar trend, i.e., the values improved from +413 mV (recrystallized) to +495 mV_SCE_ after 50 h of aging, indicating increased resistance to pitting corrosion. However, a drastic drop to +5 mV_SCE_ after 1 h of aging indicates vulnerability to chloride-induced breakdown during early thermal exposure, further substantiating the transient LOP behavior.^27^ The subsequent rise in *E*_pit_ with aging duration reinforces the alloy’s capacity for ROP and enhanced localized corrosion resistance over time.

The stability of the passive layer can also be evaluated through Δ*E = E*_pit_ *– E*_corr_. The recrystallized sample showed a high Δ*E* of 718 mV, indicating a robust passive film. This parameter dropped sharply to 265 mV after 1 h of aging, consistent with a critical LOP episode, then improved again to 620 mV after 50 h, highlighting the alloy’s ability to re-establish stable passivation upon extended thermal exposure.

The cyclic polarization curves demonstrate that the Ni_60_Cr_15_Co_15_Ti_5_Al_2.5_Nb_2.5_ HEA displays a complex corrosion response governed by heat treatment. While short aging durations may disrupt passivation and induce temporary LOP, prolonged aging consistently promotes ROP, characterized by more positive *E*_corr_, lower *i*_corr_ and *i*_pass_, and higher *E*_pit_ and Δ*E* values. This highlights the potential of post-processing heat treatments to optimize the electrochemical durability of HEAs.

These observations collectively suggest that prolongated thermal exposure enhances corrosion resistance through ROP. Therefore, an optimal heat treatment window exists where the alloy achieves maximal passivation and corrosion resistance.

The pitting potential (*E*_pit_), which indicates the onset of localized corrosion due to passive layer breakdown, also showed improvements with heat treatment. The *E*_pit_ of the recrystallized sample was +413 mV_SCE_, which increased to +495 mV_SCE_ after 50 h of aging, suggesting better resistance to pitting corrosion. However, a significant drop in *E*_pit_ to +5 mV_SCE_ after 1 h of aging indicates a pronounced LOP, where the alloy becomes more susceptible to localized attack. The recovery of *E*_pit_ with prolonged aging further underscores the occurrence of ROP and the eventual stabilization of the passive film.

Finally, the difference between the *E*_pit_ and *E*_corr_ (protection potential) reflects the stability of passivation. In the recrystallized sample, Δ*E* is 718 mV, indicating a relatively stable passive layer. However, after 1 h of aging, Δ*E* drops to 265 mV, suggesting a significant LOP. With 50 h of aging, Δ*E* improves to 620 mV, demonstrating ROP.

The SEM images in Figure 5 reveal characteristic corrosion pits formed on the Ni_60_Cr_15_Co_15_Ti_5_Al_2.5_Nb_2.5_ alloy surface after cyclic polarization. Despite the electrochemical indicators pointing to enhanced passivity after extended aging (e.g., reduced *i*_corr_, increased *E*_pit_, and improved Δ*E*), the presence of corrosion pits highlights a critical aspect of the alloy’s passivation response: localized breakdown remains possible even under apparently favorable conditions.

**Figure 5:**
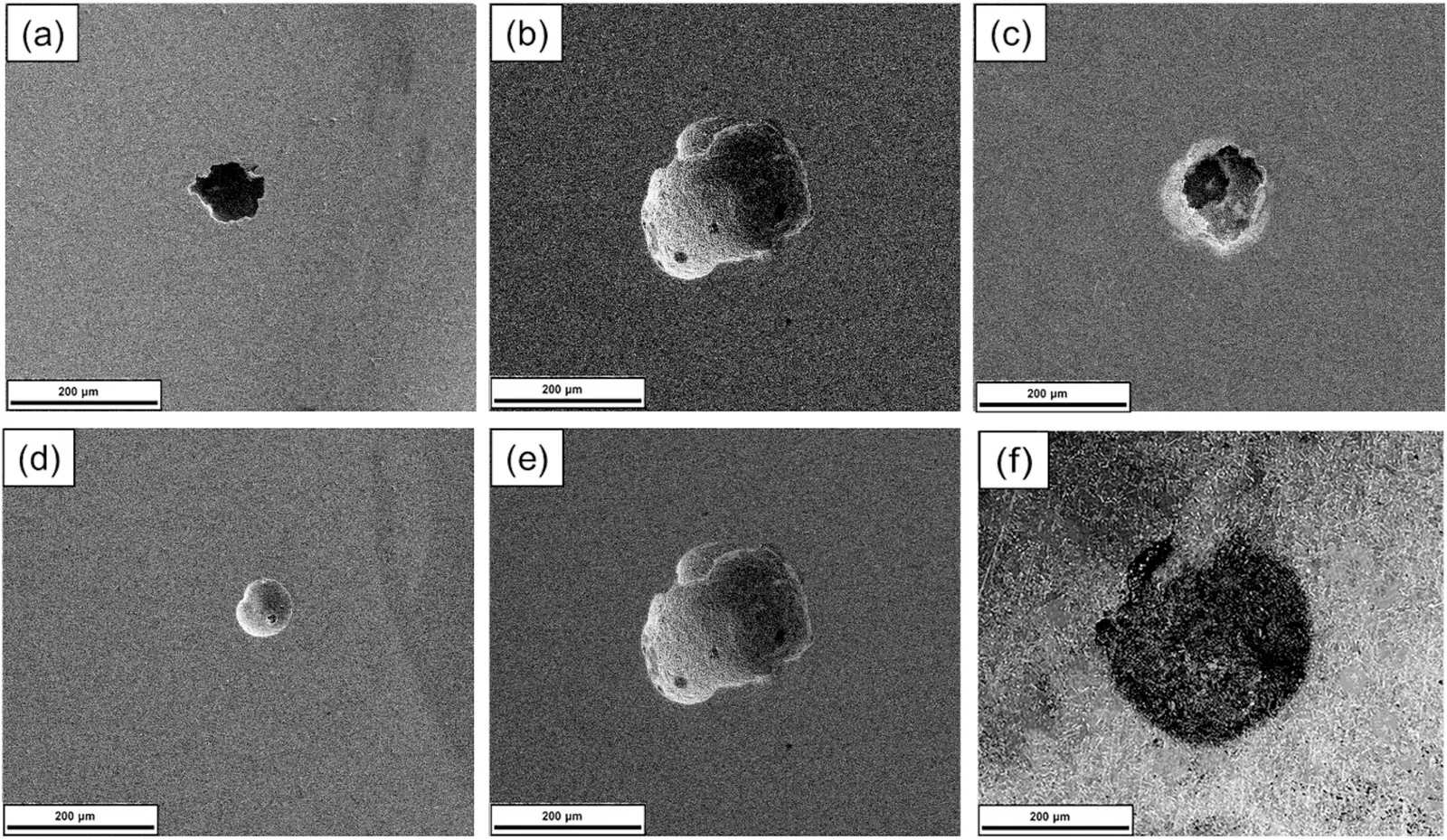
SEM images in BSE mode showing corrosion pits on the Ni_60_Cr_15_Co_15_Ti_5_Al_2.5_Nb_2.5_ alloy after thermal aging at 750 °C, surface after cyclic polarization in 3.5 wt% NaCl solution. (a) recrystallized, (b) aged for 15 min, (c) aged for 1 h, (d) aged for 10 h, (e) aged for 30 h, and (f) aged for 50 h.

This observation suggests that although prolonged aging promotes the formation of a denser and more protective passive film, likely enriched in Cr_2_O_3_ and Nb_2_O_5_, this layer may not be fully immune to localized mechanical or electrochemical instabilities. Such instabilities could arise from local compositional heterogeneities at the γ/γ′ or γ/η interfaces, or from residual stress fields associated with the coarsening of precipitates.

Moreover, the correlation between pit formation and the microstructural evolution suggests that localized zones depleted in protective elements (e.g., Cr, Nb), possibly due to preferential partitioning into precipitates or adjacent matrix regions, could act as preferential sites for passive film rupture. Previous studies have reported that coarse or incoherent secondary phases, such as overgrown η precipitates, may serve as microgalvanic couples or stress concentrators, facilitating localized attack even in systems with otherwise robust general corrosion resistance.^16,21,23^

Therefore, the pits observed after 50 h aging reinforce the hypothesis that while aging recovers the overall corrosion resistance through passive film stabilization, excessive thermal exposure may introduce vulnerabilities due to microstructural overaging effects. This finding underscores the importance of defining a precise thermal treatment window that maximizes both the protective oxide film integrity and the microstructural stability, minimizing the risk of localized passivity breakdown under service conditions.

The electrochemical analysis reveals that the Ni_60_Cr_15_Co_15_Ti_5_Al_2.5_Nb_2.5_ alloy suffers an initial LOP after short heat treatments, as reflected by the lower potentials and higher current densities. However, with extended aging, the alloy experiences a ROP, demonstrated by the recovery of the electrochemical parameters, including increasing of *E*_corr_, decreasing of *i*_corr_, enhancing of *E*_pit_, and the achievement of extended stability of the passive window upon anodic polarization.

The impedance data for the Ni_60_Cr_15_Co_15_Ti_5_Al_2.5_Nb_2.5_ alloy were analyzed to understand the behavior of LOP and ROP upon heat treatment. Key impedance parameters, including *Q* (capacitance-like response related to the passive layer), α (dispersion factor), *R*_Ω_ (ohmic resistance), *R*_p_ (polarization resistance), and *C*_eff_ (effective capacitance), were considered, as shown in Figure 6a–c by linear regression considering n-Voigt elements, and Figure 6d–f by electrical equivalent circuit. Table 3 details the values of the parameters from considering a linear regression with n-Voigt elements to the experimental EIS data using the Measurement Model and the electrical equivalent circuit approaches.

**Table 3:** (i) Electrochemical Impedance Parameters Obtained from (i) Fitting the Experimental EIS Data Using a Randles Electrical Equivalent Circuit (EEC) Model and (ii) Linear Regression Analysis Based on the Measurement Model Approach, Incorporating n-Voigt

(i) Electrical Equivalent Circuit Approach [Randle’s Modified Model]					
			CPE^a^		
sample	*R*_Ω_ (Ω·cm^2^)	*R*_p_ (kΩ·cm^2^)	*Q* (μFs^α–1^·cm^–2^)	α	χ^2^ (10^–3^)
Recrystallized	102.2 ± 0.5	930 ± 18	11.0 ± 0.2	0.916 ± 0.001	0.65
Aged 15 min	95.1 ± 0.3	959 ± 14	7.4 ± 0.4	0.911 ± 0.001	1.58
Aged 1 h	112.2 ± 0.6	277 ± 12	9.9 ± 0.7	0.926 ± 0.002	1.94
Aged 10 h	102.1 ± 0.6	890 ± 17	10.4 ± 0.6	0.896 ± 0.001	0.97
Aged 30 h	109.3 ± 0.7	1497 ± 33	6.0 ± 0.4	0.812 ± 0.001	0.56
Aged 50 h	99.94 ± 0.4	1021 ± 17	8.4 ± 0.5	0.931 ± 0.001	1.16

(ii) Linear Regression Approach (Measurement model, n-Voigt Elements)					
sample	*R*_Ω_ (Ω·cm^2^)	*R*_p_ (kΩ·cm^2^)	*C*_eff_ (μF·cm^–2^)	Voigt Number	χ^2^ (10^–3^)
Recrystallized	101.1 ± 0.1	960 ± 18	3.2 ± 0.1	8	1.78
Aged 15 min	94.1 ± 0.3	991 ± 20	2.1 ± 0.2	7	1.66
Aged 1 h	110.8 ± 1	290 ± 13	2.7 ± 0.1	7	1.01
Aged 10 h	98.5 ± 0.9	892 ± 13	6.0 ± 0.3	8	0.93
Aged 30 h	114.8 ± 0.3	1352 ± 14	7.2 ± 0.5	8	0.63
Aged 50 h	97.8 ± 0.3	1084 ± 31	2.6 ± 0.2	7	1.53

a Effective capacitance, *C*_eff (ECC)_, can be calculated from the *R*_Ω_, *R*_p_, *Q* and α values considering the Brug’s equation, *C*_eff_ = *Q* ^1/α^ (*R*_e_^–1^ + *R*_p_^–1^)^(α–1)/α^.^34^

**Figure 6:**
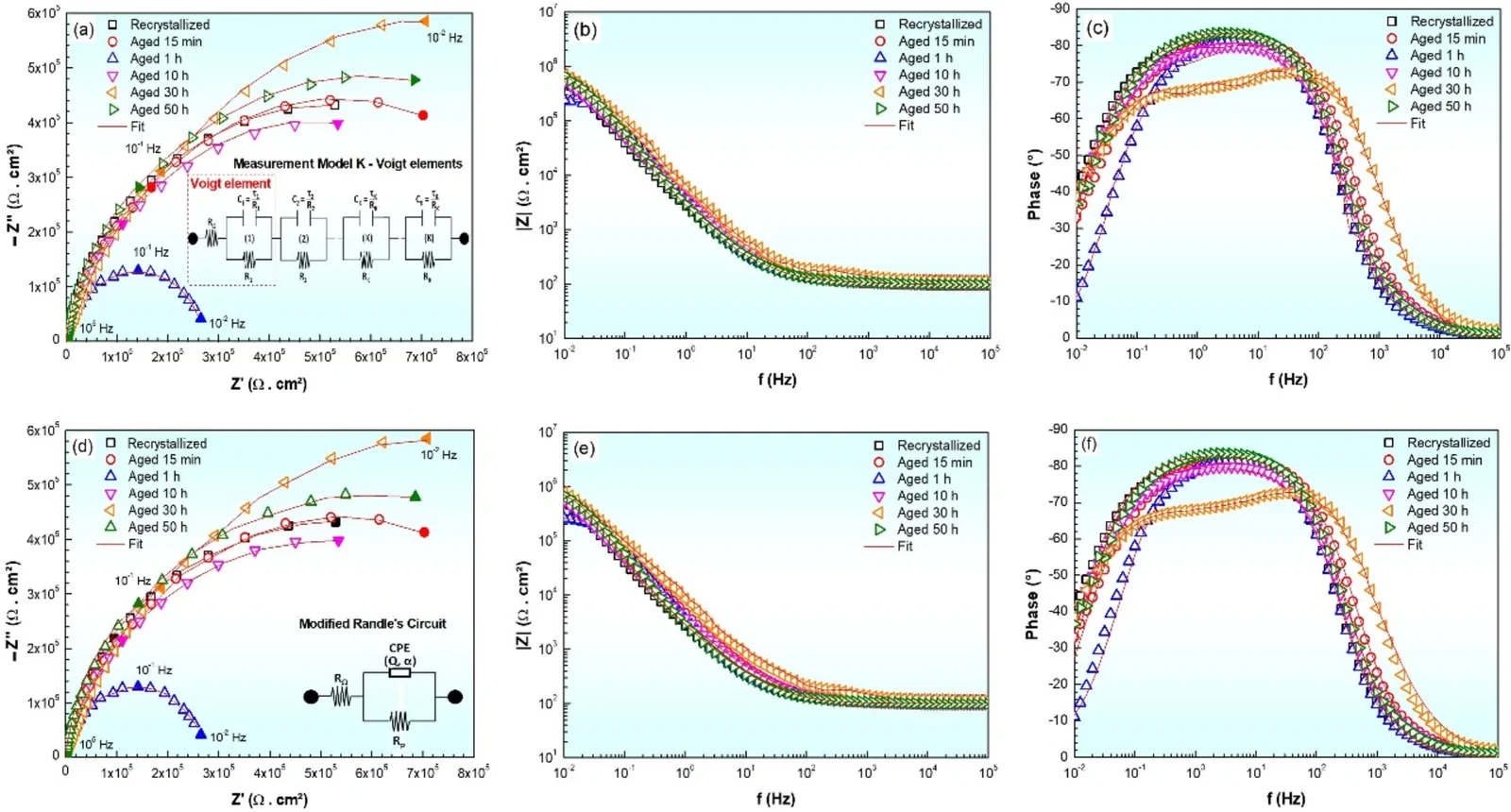
Electrochemical impedance spectroscopy (EIS) data for the Ni_60_Cr_15_Co_15_Ti_5_Al_2.5_Nb_2.5_ alloy analyzed using two different modeling approaches. (a–c) Experimental and fitted curves based on the Measurement Model employing n-Voigt elements, and (d–f) Experimental and fitted curves based on electrical equivalent circuit use; (a–d) Nyquist plots, (b–e) Bode |*Z*| plots and (c–f) Bode phase angle plots.

A typical capacitive response, characterized by truncated semicircles in the Nyquist plots, was observed across all thermal treatment conditions, confirming the presence of a passive oxide film on the alloy surface. However, the electrochemical protectiveness of this film varied significantly with the duration of heat treatment. Specifically, the sample aged for 1 h exhibited the smallest semicircle diameter, indicating the lowest polarization resistance and suggesting a transient degradation or instability in the passive layer. With prolonged aging, a notable recovery, and even enhancement, of polarization resistance was observed, implying a progressive improvement in the stability and integrity of the passive film over time.

In the ECC approach the electrochemical impedance behavior of the Ni_60_Cr_15_Co_15_Ti_5_Al_2.5_Nb_2.5_ alloy was modeled using a modified Randles-type analog circuit, which is commonly employed to describe interfacial processes in high-entropy alloys (HEAs).^28–33^ The circuit comprises the solution resistance *R*_Ω_ in series with a constant phase element (CPE), which is in parallel with a polarization resistance (*R*_p_), expressed as *R*_Ω_ *+* CPE*/R*_p_. This configuration effectively captures the interfacial electrochemical processes occurring at the alloy–electrolyte boundary, as commonly observed in high-entropy alloys (HEAs). The *R*_Ω_ component represents the resistance of the electrolyte, while *R*_p_ reflects the polarization resistance, often linked to the protective properties of the passive film together with the charge transfer resistance. A constant phase element was employed instead of an ideal capacitor to account for the nonideal dielectric behavior typically observed in real systems, which arises due to surface heterogeneities or inhomogeneous current distribution. The CPE is characterized by two parameters: *Q*, the pseudo-capacitance, and α, an empirical exponent (0 < α ≤ 1) that quantifies the deviation from ideal capacitive behavior. When α approaches 1, the CPE behaves more like an ideal capacitor, indicating a more homogeneous and stable interface.

Across all thermal conditions, the EEC and Measurement Model approaches exhibit consistent trends in the variation of polarization resistance (*R*_p_) and effective capacitance (*C*_eff_), reinforcing the interpretation of corrosion resistance evolution. However, the absolute values differ between the two approaches due to inherent modeling assumptions. For instance, the EEC-derived *R*_p_ values are slightly lower than those obtained from the n-Voigt-based Measurement Model, which more finely resolves overlapping relaxation processes due to its distributed element structure.

In the recrystallized state, both models identify a relatively high *R*_p_ (930–960 kΩ·cm^2^) and moderate *C*_eff_ (3.2–5.9 μF/cm^2^), indicative of a stable passive film. After 1 h of aging, a pronounced decline in *R*_p_ (down to ∼277–290 kΩ·cm^2^) and modest increase in *C*_eff_ reflect a temporary degradation in passivation, captured by both models. The corresponding increase in χ^2^ values (1.94 and 1.01) further suggests a more complex and possibly unstable interfacial condition at this stage. These electrochemical trends are consistent with the polarization data, which show elevated *i*_corr_ (2.7 μA/cm^2^), a low *E*_pit_ (+5 mV_SCE_), and a very negative *E*_prot_ (−227 mV_SCE_), pointing to poor resistance against localized corrosion. This is likely due to localized nickel depletion and the presence of incoherent eta particles acting as corrosion initiation sites.^21,23^

With extended aging (30 h), both models capture the peak of corrosion resistance, albeit with varying resolution: the EEC model reports the highest *R*_p_ (1497 kΩ·cm^2^) and the lowest *C*_eff_ (1.09 μF/cm^2^), while the Measurement Model yields slightly lower *R*_p_ (1352 kΩ·cm^2^) but a higher *C*_eff_ (7.2 μF/cm^2^), a clear indication of ROP. Despite these discrepancies, both approaches converge in identifying this condition as electrochemically optimal, corroborated by the lowest χ^2^ values (0.56 and 0.63, respectively), which attest to the accuracy of the model fits at this stage. Supporting this, partial recovery of both impedance and polarization parameters was already observed after 10 h of aging, suggesting that beneficial features that recover the corrosion resistance are initiated during intermediate thermal exposure. Indeed, at 30 h the alloy reaches an electrochemically optimal condition. The improvement in passivation is corroborated by polarization results. After 30 h of aging, the alloy exhibited excellent electrochemical performance: low *i*_corr_ (0.9 μA/cm^2^), a high *E*_pit_ (+480 mV_SCE_) and a positive *E*_prot_ value (+289 mV_SCE_).

At 50 h, both models detect a moderate decline in *R*_p_ and an increase in *C*_eff,_ signaling the onset of passive film destabilization, a signal of a secondary LOP process after prolonged treatment. Although the corrosion current density is low at this stage (0.07 μA/cm^2^), the negative *E*_pro*t*_ (−41 mV_SCE_) suggests the passive layer is unstable and repassivation is not effectively achieved following pit initiation. Interestingly, while the EEC model reports a noticeable increase in *C*_eff_ (4.94 μF/cm^2^), the Measurement Model suggests a decline (2.6 μF/cm^2^), potentially due to differing sensitivities to surface heterogeneity versus time-constant distributions. This discrepancy underscores the importance of a dual-model strategy: while the EEC model excels at capturing global electrochemical features, the Measurement Model provides a more nuanced breakdown of dynamic interfacial behavior via its Voigt decomposition, with Voigt numbers remaining relatively constant (7–8) across all treatments.

The electrochemical data revealed typical passive behavior in Ni-based alloys. The corrosion current density (*i*_corr_) was very low, on the order of 10^–6^ A/cm^2^, consistent with strong material passivity.^35^ The polarization curves exhibited a wide passive region, with passivation current densities (*i*_pass_) around 10^–5^–10^–6^ A/cm^2^ and a pitting potential (*E*_pit_) significantly above the corrosion potential (*E*_corr_), indicating good pitting resistance.^35^ These features are consistent with the formation of a mixed NiO/Cr_2_O_3_ passive film. Previous studies show that the inner Cr_2_O_3_ layer provides most of the protection, rupturing at about 0.75 V, whereas the outer NiO only breaks down at much higher potentials (around 0.90 V). Thus, the presence of Cr in the alloy is crucial for passive film stability and explains the observed resistance.^29,36,37^

Microstructurally, aging promoted both grain growth and increased η (Ni_3_(Ti,Nb)) precipitation. These precipitates consume Nb and Ti from the γ/γ′ matrix and can introduce local heterogeneities, but they also enrich the matrix in Cr. In Ni–Cr alloys, Cr poor precipitates effectively leave the surrounding matrix relatively Cr rich, favoring Cr_2_O_3_ passive film formation.^31,35^ Similar effects in niobium-stabilized stainless steels show that Nb enhances corrosion resistance by retaining free Cr via carbide formation. Conversely, compositional differences between η and γ regions can create local galvanic microcells; the observed banded attack suggests microgalvanic corrosion in segregated areas, as reported in duplex steels where Cr depleted zones act as anodes.^11,28,35^

From a thermodynamic perspective, passive film formation is driven by the low Gibbs free energies of formation for Cr_2_O_3_ (Δ*G* ^0^ ∼ −540 kJ·mol^–1^) and Nb_2_O_5_ (Δ*G* ^0^ ∼ −740 kJ·mol^–1^), making these oxides highly stable in oxidizing environments.^38^ Their spontaneous formation minimizes the system’s free energy and promotes a continuous protective barrier on the metal surface. Kinetically, film quality and thickness depend on the diffusion of film-forming elements (especially Cr and Nb) to the metal–electrolyte interface. This underlines the critical role of prolonged thermal aging (30–50 h), which enhances elemental redistribution and fosters a denser, more homogeneous passive film. The decrease in *i*_corr_, increase in polarization resistance (*R*_p_), and noble shift in *E*_corr_ after aging indicate that anodic reactions become diffusion limited, likely due to fewer film defects and reduced exposed active area.

Furthermore, the passivation recovery observed after 10–50 h can be understood via the point defect model (PDM), which states that passive film stability depends on the balance between cationic and anionic vacancy generation and migration through the oxide layer.^39,40^ Films with high defect densities allow intense ionic fluxes, accelerating film growth or local breakdown. Thermal aging reduces segregation and stabilizes the microstructure, decreasing defect formation and stabilizing film growth. Consequently, the system reaches a thermodynamically more stable and kinetically less active state, reflected in increasing *E*_pit_ and protection potential (*E*_prot_) and in reduced *i*_pass_.

The electrochemical impedance response of the alloy was analyzed considering both the electrochemical equivalent circuit approach (EEC) and the Measurement Model based on n-Voigt elements. While EEC captures the dominant interfacial processes through a single distributed capacitance, the n-Voigt decomposition resolves the system into multiple parallel RC elements, each associated with a relaxation constant. The need for seven to eight Voigt elements in this case reflects the high degree of heterogeneity in the passive film, which can be rationalized as a resistivity gradient extending from the alloy/passive film interface to the film/electrolyte boundary. In this scenario, the oxide layer is more realistically represented as a stack of sublayers, each contributing to the overall capacitive and resistive behavior of the film. This layered representation, the n-Voigt model, explains the overlapping relaxation processes detected in the spectra, particularly under aging conditions, where barrier protection is enhanced by the incorporation of Cr and Nb rich oxides. Therefore, combining a single-CPE EEC model with the distributed n-Voigt approach provides complementary insights, the former identifying the dominant resistance to charge transfer and the latter resolving the complex spectrum of relaxation times associated with the passive layer.

These findings are consistent with the behavior of other Cr rich Ni based alloys and HEAs, where corrosion resistance was attributed to the formation of continuous Cr_2_O_3_/Nb_2_O_5_ films and suppression of deleterious phases through controlled heat treatments. Thus, the data in this work not only validate these mechanisms but also highlight the importance of designing thermal treatments that optimize the diffusion and redistribution of active elements to maximize the electrochemical performance of complex alloys in aggressive environments.^31,33,35^

## 3 Conclusions

The present work led to conclusions highlighting how thermal aging modulates the microstructure and stability of the passive film of the Ni_60_Cr_15_Co_15_Ti_5_Al_2.5_Nb_2.5_ alloy, providing clear guidelines for controlling its corrosion performance in chloride rich environments:

1. Thermal aging at 750 °C revealed a strong interplay between microstructural evolution and passivation in the Ni_60_Cr_15_Co_15_Ti_5_Al_2.5_Nb_2.5_ alloy.
2. Short exposures of 15 min to 1 h induced a transient loss of corrosion resistance, as evidenced by an increase in *i*_corr_ to 2.4 μA/cm^2^, and a reduction in *E*_pit_ by more than 200 mV.
3. With extended aging from 10 to 50 h, elemental redistribution favored the development of a dense, stable passive film, leading to an increase in *R*_p_ from 28 to over 150 kΩ·cm^2^ and a reduction in *i*_corr_ by more than an order of magnitude. The most protective condition was achieved between 30 and 50 h, when the oxide stability and microstructural state reached an optimal balance, minimizing susceptibility to localized attack.
4. These results demonstrate that corrosion performance in high-entropy alloys can be effectively tailored through precise control of aging parameters, enabling their reliable deployment in chloride-rich service environments.

## 4 Materials and Methods

### 4.1 Computational Design

The compositional design of a new superalloy was carried out using the High-Throughput Calculations (HTC) module of the Pandat software, in conjunction with the PanNi2022 thermodynamic database. Simulations were conducted under equilibrium solidification conditions for the Cr–Co–Ni–Al–Ti–Nb system, with compositional limits defined based on ranges typically observed in commercial wrought superalloys.

For each element, a specific molar concentration range and increment were assigned as input to the HTC model, where the increment (Δ*x*) corresponds to the step size used in the combinatorial calculations across the defined compositional space. Specifically, the following ranges and step values were considered: Al (0–10 mol %, Δ*x* = 2.5), Co (0–20 mol %, Δ*x* = 5.0), Cr (10–25 mol %, Δ*x* = 2.5), Nb (2.5–7.5 mol %, Δ*x* = 2.5), Ti (0–5 mol %, Δ*x* = 2.5), and Ni (30–70 mol %, with no increment, as it was adjusted implicitly to satisfy the overall compositional balance). The selection of suitable alloy candidates was based on three thermodynamic criteria applied to the HTC output: (i) a single-phase FCC field at high temperatures and solvus temperature not exceeding 1100 °C, (ii) a stability range in which only FCC, L1_2_ (γ′), and D0_22_ (γ″) phases coexist between a minimum temperature (*T*_min_ ≤ 650 °C) and a maximum temperature (*T*_max_ ≥ 800 °C), and (iii) a maximum volume fraction of the combined L1_2_ and D0_22_ phases limited to 0.5. The boundary conditions, synthesis protocol, and computational details assigned to each element were based on previous work by the same research group, available at ref.^38^

These constraints were designed to ensure processability during hot working above the solvus, to align with the typical operating temperature range of nickel-based superalloys, and to maintain γ′/γ″ fractions within the optimal strengthening range for polycrystalline materials. After filtering, the optimal alloy composition determined was Ni_60_Cr_15_Co_15_Ti_5_Al_2.5_Nb_2.5_ (in at%).

### 4.2 Alloy Production

The Ni_60_Cr_15_Co_15_Ti_5_Al_2.5_Nb_2.5_ alloy was manufactured by induction melting furnace, model VIP 50–30R Power Track with commercially pure raw materials, resulting in a 3 kg ingot. A segment of the alloy was remelted by arc melting under argon atmosphere and homogenized at 1200 °C for 20 h. After homogenization, a sample was cold rolled in multiple stages from a thickness of 3 mm and reduced to approximately 1 mm. The recrystallization of the sample was performed at 1150 °C for 1 h and water quenched. After these recrystallization processes, the samples were aged at 750 °C in five different time intervals: 15 min, 1 h, 10 h, 30 and 50 h.

### 4.3 Characterization Methods

The phase analysis of the samples was carried out using X-ray diffraction (XRD, Anton Paar) in transmission mode with a molybdenum (Mo) target (λ = 0.7093 Å). Diffraction patterns were collected over the 2θ range of 5° to 50°, with a step size of 0.02° and a scan rate of 0.5 s per step. Microstructural observations were conducted using scanning electron microscopy (FEG-SEM, TESCAN Mira) in backscattered electron (BSE) mode, as well as transmission electron microscope (TEM, Talos F200x G2). The samples were ground with SiC papers up to 1500 grit, polished with 1 μm Al_2_O_3_ suspension, and finished with colloidal silica.

The corrosion behavior of the alloy was investigated using cyclic polarization and electrochemical impedance spectroscopy (EIS) in a 3.5 wt % NaCl solution, prepared from high-purity NaCl (>99%) and deionized water. The cyclic polarization tests were performed using a standard three-electrode cell configuration connected to a Gamry 600+ potentiostat. The working electrode (WE) was the alloy sample subjected to different thermal aging conditions, with an exposed area of 0.3 cm^2^. A platinum grid was used as the counter electrode (CE), and a saturated calomel electrode (SCE) served as the reference electrode.

Prior to testing, all samples were stabilized at open circuit potential (OCP) for 1 h. The cyclic polarization scan was initiated at −300 mV versus OCP, swept anodically at a scan rate of 1 mV/s^1^ until reaching a current density of 10^–2^ A/cm^2^, followed by a reversal of the potential in the cathodic direction to complete the loop. The corrosion potential (*E*_corr_) and corrosion current density (*i*_corr_) were determined by extrapolating the linear Tafel region of the cathodic branch. The pitting potential (*E*_pit_) was taken as the potential at which a sharp increase in current occurred, and the protection potential (*E*_*p*rot_) was identified during the reverse scan as the point at which the current density sharply dropped, indicating repassivation.

EIS was conducted to complement the polarization data and gain further insights into the electrochemical properties of the passive film. After 1 h of OCP stabilization, impedance measurements were taken using a ± 10 mV sinusoidal perturbation over a frequency range from 10^5^ to 10^–2^ Hz, with 10 data points per frequency decade. As with the polarization tests, three replicates were analyzed per tempering condition to ensure data reproducibility.

Two complementary approaches were employed to analyze the electrochemical impedance spectroscopy (EIS) data, each offering unique advantages for interpretation. The first method utilized the Measurement Model developed by Watson and Orazem,^41^ which applies a linear regression framework to fit the impedance response. This model consists of an ohmic resistance (*R*_Ω_) in series with multiple Voigt elements, where each element represents a parallel resistor–capacitor (*R–C*) pair. Through this approach, key electrochemical parameters including polarization resistance (*R*_p_), ohmic resistance (*R*_Ω_), and effective capacitance (*C*_eff_) were extracted. The time constant for each Voigt element is defined as τ*_i_* = *R_i_* ·*C_i_*, and the capacitance as *C*_*i*_ = τ_*i*_/*R_i_*. The effective capacitance (*C*_eff_) was determined from the high-frequency limit (ω → ∞) of eq 1, yielding eq 2, while the polarization resistance (*R*_p_) was obtained from the low-frequency limit (ω → 0) of eq 1, as expressed in eq 3

1 $$Z=R_{\Omega }+\left(C\right)+\sum_{i=1}^{n}\frac{R_{i}}{1+j\omega \tau_{i}}$$

2 $$\frac{1}{C_{\mathrm{eff}}}=\sum_{i=1}^{n}\frac{1}{C_{i}}$$

3 $$R_{p}=R_{\Omega }+\sum_{i=1}^{n}R_{i}$$

To facilitate comparison with existing literature, an equivalent electrical circuit (EEC) model was simultaneously employed using EC-Lab software (BioLogic). While the EEC model enables direct correlation with published data for similar alloy systems, the Measurement Model offers superior physical interpretability and more reliable extraction of *C*_eff_ and *R*_p_ values through its consideration of the surface’s heterogeneity.
